# One- and Two-Year Efficacy of Resin Infiltration and Remineralization for the Treatment of Initial Proximal Caries

**DOI:** 10.3390/jfb16070242

**Published:** 2025-07-01

**Authors:** Veselina Todorova, Ivan Filipov

**Affiliations:** Department of Operative Dentistry and Endodontics, Faculty of Dental Medicine, Medical University of Plovdiv, 4000 Plovdiv, Bulgaria; filipov@abv.bg

**Keywords:** resin infiltration, remineralization, initial proximal caries

## Abstract

Proximal caries presents diagnostic and therapeutic challenges. Recent understanding of the etiology and pathology of dental caries has led to the adoption of non-invasive and/or minimally invasive approaches in the early stages of caries lesions. This clinical study aimed to compare the efficacy of resin infiltration and remineralization in the treatment of initial proximal caries lesions over a 1- and 2-year follow-up period. The study involved 47 patients aged between 18 and 38 years. Patients were clinically examined and underwent bitewing radiography to detect at least three initial proximal caries lesions. Each detected lesion (180 in total) was randomly assigned to one of three treatment groups: (1) resin infiltration with Icon Proximal Infiltrant (DMG); (2) remineralization with Clinpro White Varnish (3M); and (3) a control group receiving no treatment. One year after treatment, caries progression was found in 30 lesions (16.6%) with the following distribution across the three treatment groups: 2/60 (3%) in the infiltration group; 11/60 (18%) in the remineralization group; 17/60 (28.30%) in the no treatment control group with a significant statistical difference between the groups (*p* = 0.001). In terms of lesion depth, 12 (11%) out of 106 E2 lesions progressed and 18 out of 74 (24%) D1 lesions progressed, with a significant difference (*p* = 0.037). Two years after treatment, five new lesions were found to have progressed (one E2 and four D1), distributed as follows: 0% in the infiltration group, 3.6% in the remineralization group, and 5% in the control group. In conclusion, resin infiltration exhibited the lowest percentage of progressed lesions and could be considered a reliable, non-invasive treatment for initial proximal caries.

## 1. Introduction

Minimal intervention is the main goal of contemporary caries therapy. There has been a paradigm shift in cariology from the conventional approach of “drill and fill” to a new philosophy of “heal and seal” [1]. Caries is a relatively slowly progressing disease, which allows it to be treated and arrested at any time and requires a rethinking of the early invasive intervention [2,3]. Recent understanding of the etiology and pathology of dental caries has led to the adoption of non-invasive and/or minimally invasive approaches in the early stages of caries lesions [4].

Proximal caries presents both diagnostic and therapeutic challenges. Difficult access to caries lesions can prevent their early detection and treatment [5]. Advances in technology have led to the development of numerous devices for the early detection of caries, enabling its diagnosis and proper treatment at the optimal time.

General strategies for managing initial proximal caries until recently have focused on either remineralization or invasive treatment. Traditional preventive measures aim to increase remineralization by applying fluoride varnishes, as well as improving the patient’s oral hygiene and dietary habits [6]. Unfortunately, in many cases, these measures only slow down the progression of caries lesions and are unable to arrest them [1,7]. Furthermore, the potential of fluoride and other remineralizing agents to protect the enamel is restricted to the outer ~30 µm of the tooth [8]. Lack of patient cooperation can also contribute to lesion progression and enamel cavitation [9]. Once a cavity has formed, non-invasive measures are ineffective, and the demineralization process continues [7]. The presence of cavitation is an indication for an operative intervention. Particularly on proximal surfaces, access to the caries lesion requires the removal of large areas of healthy enamel, even when minimally invasive preparation techniques are used [10,11]. An alternative therapy for arresting initial caries is the infiltration with light-curing resins. The aim of resin infiltration is to seal the pores in the lesion, thus preventing the penetration of acids and mechanically stabilizing the weak demineralized enamel [12]. For incipient enamel lesions on smooth surfaces, the application of infiltrating resins can stop progression or delay the first operative intervention with several years [3,12]. Resin infiltration fills the existing therapeutic gap between pure prophylaxis and operative treatment and completely complies with the concept of minimal intervention in dentistry. The method is new and promising, but more long-term clinical studies are needed to establish it and impose it for routine use in the treatment of proximal caries.

While previous studies have evaluated the effectiveness of resin infiltration and placebo group or fluoride-based remineralization techniques separately, few have offered a direct, clinical comparison of these modalities under controlled clinical conditions and over a prolonged follow-up period. Clinical trials in permanent teeth have revealed a higher efficacy of the infiltration technique compared to varying non-invasive interventions alone [12,13,14,15,16,17]. A Cochrane meta-analysis concluded that resin infiltration is significantly more effective than non-invasive professional treatment and/or oral health advice but underlined that long-term results are necessary [18]. The aim of the present study was to assess the clinical efficacy of resin infiltration for the treatment of initial proximal caries lesions for a follow-up period of one and two years. It was hypothesized that the radiographic lesion progression of proximal lesions in the infiltration group would be significantly reduced compared with that in the remineralization and the control group (no therapy). The results of the study could provide a comprehensive understanding of how micro-invasive and non-invasive treatments perform in managing initial proximal caries lesions.

## 2. Materials and Methods

The study was approved by the Institutional Ethics Committee of Medical University of Plovdiv, Bulgaria (protocol code number 7/date of approval 1 October 2020).

The inclusion criteria were: (1) the acquisition of informed consent; (2) the identification of a minimum of three non-cavitated proximal caries lesions, radiographically confirmed to extend to the inner half of the enamel or the outer third of the dentin for each patient; and (3) non-cavitated lesions.

The exclusion criteria comprised: (1) the absence of informed consent; (2) fewer than three radiographically visible initial proximal lesions; (3) the presence of cavitation; (4) pregnancy, contraindicating radiography; and (5) allergy to monomers.

From 134 screened individuals, 47 patients aged between 18 and 38 years met the inclusion criteria, gave their informed consent, and were enrolled in the study. For each patient, one group of three initial caries lesions (36 patients), two groups of three lesions (9 patients), or three groups of three lesions (2 patients) of proximal caries with radiographic scores E2 (inner half of enamel) or D1 (outer third of dentin) were chosen by the investigator based on site of lesion occurrence. From each three lesions, one lesion was allocated to infiltration treatment, one to remineralization treatment and one to the control (no treatment) group, respectively. A total of 180 initial proximal caries lesions with a radiographic depth ranging from E2 to D1, divided into three treatment groups of 60 lesions each, were included in the study. The radiographic depth of the lesions was evaluated before treatment and 1 and 2 years after treatment.

### 2.1. Sample Size Justification and Randomization

A formal sample size calculation was not conducted prior to study initiation. Instead, the sample size of 180 proximal caries lesions (distributed among 47 adult patients) was based on precedent from comparable randomized controlled clinical studies evaluating micro-invasive treatments [10,11], which demonstrated meaningful intergroup differences with lesion counts between 50 and 60 per group. The allocation of 60 lesions per treatment group was deemed sufficient to detect clinically meaningful differences in progression rates across intervention arms, while allowing subgroup analyses by lesion depth (from E2 and D1).

Allocation of the 180 lesions into three equal groups (*n* = 60) was performed using computer generated randomly permuted blocks (generated by a third person, sealed in envelopes) [10], ensuring balanced group sizes. In this way, allocation was concealed from the radiographic evaluator, who was blinded to the treatment group during follow-up assessment.

### 2.2. Patients Examination

Patients underwent a clinical examination and had two initial bitewing radiographs taken to detect at least three proximal caries lesions in their mouths. The bitewing radiographs were digital, using a phosphor-sensitive plate (Dűrr Dental SE, Bietigheim-Bissingen, Germany) and an Icon X-ray Holder (DMG, Hamburg, Germany) fixed with O’Bite silicone impression material (DMG, Hamburg, Germany). The X-ray device was Planmeca (Helsinki, Finland). The bitewing radiographs were assessed under standard conditions on a computer screen (Dűrr Dental computer program).

The patient’s caries risk was assessed. The gingival status was recorded, as well as bleeding from the papillae after gentle air-drying as an indirect indicator of lesion activity. Wedges were placed between the teeth for temporary separation. Visual assessment of the lesions according to the ICDAS criteria, as well as assessment of lesion activity according to the criteria by Ekstrand et al. was performed [19].

Each detected caries lesion was randomly assigned to one of the following treatment groups:

1. First group—resin infiltration with Icon Proximal Infiltrant (DMG Dental, Hamburg, Germany).
2. Second group—remineralization with Clinpro White Varnish (3M Oral Care, St. Paul, MN, USA).
3. Third group—control group with no treatment.

### 2.3. Rationale for Treatment Materials Selection

Icon Proximal Infiltrant (DMG, Hamburg, Germany) and Clinpro White Varnish (3M Oral Care, St. Paul, MN, USA) were selected based on their mechanisms of action and evidence-based efficacy, allowing a meaningful comparison between micro-invasive and non-invasive approaches for the treatment of initial caries lesions.

Resin infiltration was selected for this study based on its favorable physical properties and well-documented clinical performance [18,20]. The material used (TEGDMA) is a low-viscous resin with a high penetration coefficient (more than 100 cm/s), which allows it to deeply penetrate and infiltrate demineralized enamel and early dentin lesions through capillary action [21]. This mechanism effectively arrests caries progression by sealing the porous lesion structure and blocking diffusion pathways for acids and fermentable substrates [1,3,12]. To enhance resin penetration, the lesion surface is pre-treated with 15% hydrochloric acid, which removes the superficial pseudo-intact enamel layer and exposes the underlying lesion body, improving resin penetration by capillary forces [22]. These steps allow for thorough infiltration of the lesion, transforming a porous substrate into a mechanically stabilized, sealed structure.

Clinpro White Varnish (3M Oral Care, St. Paul, MN, USA) was selected as the remineralization agent in this study due to its bioactive composition and ease of application. The varnish contains 5% sodium fluoride (NaF) and functionalized tricalcium phosphate (f-TCP), which is designed to enhance fluoride-mediated remineralization by stabilizing calcium and phosphate ions until they are released in the oral environment [8]. Varnishes containing tricalcium phosphate or casein phosphopeptide amorphous calcium phosphate, when compared to sodium fluoride varnish, have demonstrated a similar efficacy against caries in high-caries-risk patients [23,24]. When applied to enamel surfaces, Clinpro White Varnish forms a calcium fluoride-like reservoir and facilitates mineral deposition in demineralized enamel through ion exchange, increasing enamel resistance to acid attack [25]. When f-TCP is delivered to the saliva-moisturized tooth structure, the protective barrier breaks down, allowing calcium, phosphate, and fluoride ions to become free and available for remineralization [26]. Due to its ability to flow and reach difficult to access areas, the varnish was considered appropriate for application in the interproximal areas. Clinpro White Varnish contains a specially designed resin that is practically invisible and does not compromise aesthetics. Previous studies have also implied that the varnish promotes the remineralization of the surface and subsurface enamel layers [26,27]. Clinical studies demonstrate that Clinpro White Varnish is effective in slowing enamel demineralization, particularly in high-caries-risk patients, orthodontic cases, and in preventing white spot lesions [28].

The two products in the study represent two different interventions for initial caries lesions, each with a different mechanism of action. Comparing them would provide a more thorough clinical perspective on their use in managing incipient caries.

### 2.4. Treatment Protocol

All patients underwent professional oral hygiene with a polishing brush and a fluoride-free polishing paste and dental floss.

**Figure 1:**
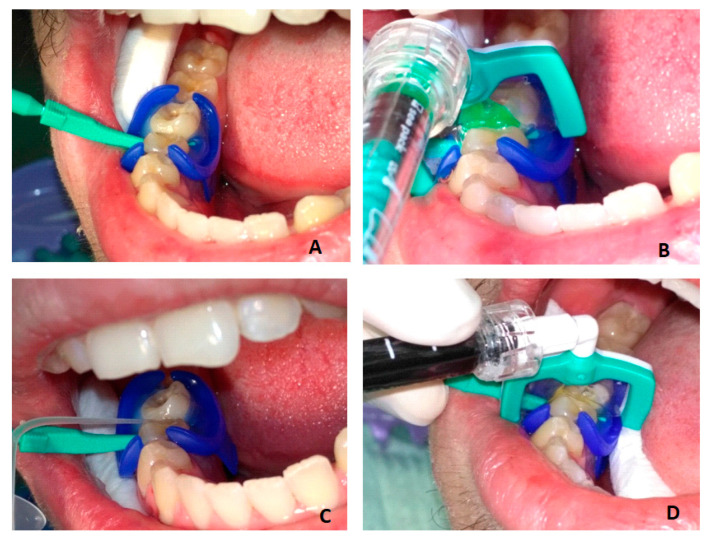
Resin infiltration: (**A**) tooth isolation and separation; (**B**) etching with 15% hydrochloric acid—Icon Etch for 2 min; (**C**) drying with pure alcohol—Icon Dry for 30 s; (**D**) infiltration with Icon Infiltrant for 3 min, light-curing and second infiltration for 1 min.

The infiltration protocol included (Figure 1):

- cleaning the tooth and isolating it with a rubber dam (MiniDam, DMG, Germany), placing a wedge (included in Icon Proximal, DMG, Germany) to separate the teeth;
- etching the tooth surface—Icon Etch (15% HCl) for 2 min;
- rinsing with water for 30 s and drying;
- application of Icon Dry (99% ethanol) for 30 s, followed by air drying;
- application of Icon Infiltrant (TEGDMA) for 3 min, removal of excess and light-curing for 40 s;
- reapplication of Icon Infiltrant for 1 min, followed by light-curing for 40 s;
- polishing.

**Figure 2:**
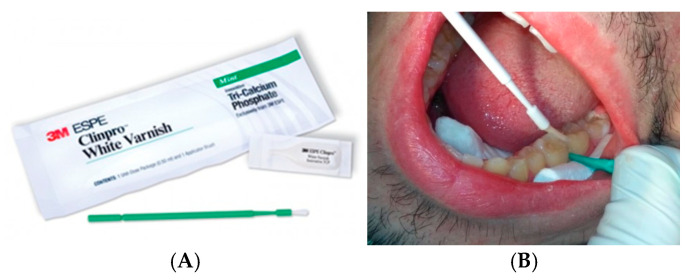
Remineralization: (**A**) Clinpro White Varnish, 3M; (**B**) interproximal tooth separation and varnish application with a brush.

Remineralization protocol: Teeth were isolated with cotton rolls and air-dried. A wedge was inserted into the interproximal space (interproximal wedge from the infiltration system—Icon Proximal). The content of the individual dose of varnish was mixed right before use (Figure 2). With the brush provided, a small amount was applied to the proximal surface (Figure 2). Patients were instructed not to brush their teeth or eat hard or sticky foods immediately after application to ensure maximum contact of the varnish with the tooth surface.

All patients were given instructions on how to improve their oral hygiene and diet and were taught to clean interdental spaces properly and regularly with dental floss.

To avoid misinterpretation of infiltrated lesions during a visit to another dentist, which could lead to premature invasive treatment, the patients were given a treatment booklet. It described the radiographic depth of the lesion of the treated tooth, as well as the date and type of treatment.

Control examinations were performed after one and two years. During the clinical examination, the patient’s caries risk was assessed again. Bitewing radiographs were taken. The radiograph holder was the same as the one used for the initial radiographs: Icon X-ray Holder (DMG, Hamburg, Germany). Each patient had their own X-ray holder. This reduced the risk of proximal surface overlap and ensured consistent positioning for radiography before treatment and at the 1- and 2-year follow-ups. The baseline to one and two-year presence or absence of caries progression was assessed using two methods: (1) radiographs were independently assessed visually; (2) radiographs were analyzed in pairs (baseline and control follow-up).

### 2.5. Statistical Analysis

Data were analyzed using IBM SPSS Statistics for Windows, version 27.0 (IBM Corp., Armonk, NY, USA). The presence of caries lesions was reported as a number (*n*) and as a relative proportion. The statistical analysis included a t-test for independent samples to compare normally distributed continuous variables, such as age; a Chi-square test to assess the association between normal or ordinal variables; and Fisher’s exact test to compare two proportions. Results were interpreted as statistically significant at *p* < 0.05.

## 3. Results

### 3.1. General Characteristics of the Studied Group of Patients

The mean age of the study group was 26.12 ± 5.72 years, ranging from 18 to 38 years. The relative share of men and women was very similar: 48.9% women (*n* = 23) and 51.1% men (*n* = 24), with no significant difference (*p* = 0.998). The mean age of female (25.47 ± 5.21 years) and male (26.75 ± 6.21 years) patients was similar, with no significant difference, *p* = 0.452.

Patients were categorized into three groups according to the degree of caries risk: low, medium, and high. Twenty patients, constituting 42.5% of the study group, were diagnosed with low risk. Eighteen patients (38%) were categorized with medium risk, and high risk was determined for the remaining nine (19.5%) patients.

### 3.2. Data on the Teeth Included in the Study

A total of 115 teeth in the upper jaw were treated, accounting for 64% of all 180 teeth included in the study. Of these, 33 teeth (18.3% of all teeth) were first premolars; 57 teeth (31.7% of all teeth) were second premolars; and 25 teeth (13.9% of all teeth) were first molars.

The remaining 65 teeth were located in the lower jaw, making up 46% of the total number. Of these, 15 (8.3% of all teeth) were first premolars ; 30 (16.7% of all teeth) were second premolars; 19 (10.6% of all teeth) were first molars; and 1 (0.6%) was a second molar. In general, the teeth in the upper jaw were predominant, and in both jaws, the number of the second premolars was the highest.

Eighty-eight proximal surfaces (49%) were mesial and 92 (51%) were distal. The distribution of the surfaces across the three experimental groups was very similar, without significant difference according to the Chi-square test (*p* = 0.915). The mesial surfaces made up 50% (*n* = 30) of the lesions treated with infiltration; 47% (*n* = 28) of those with remineralization; and 50% (*n* = 30) of the control group. Similarly, distal surfaces comprised 50% (*n* = 30) of the lesions treated with infiltration; 53% (*n* = 32) of those with remineralization; and 50% (*n* = 30) of the control group.

### 3.3. Distribution of Caries Lesions According to Depth and Treatment

Out of the 180 caries lesions, 106 (59%) were in stage E2 (reaching the inner half of the enamel), and 74 (41%) were in stage D1 (reaching the outer third of the dentin). In terms of treatment groups, the distribution of the 106 lesions with depth E2 was as follows: 35 (33%) in the infiltration group, 36 (34%) in the remineralization group, and 35 (33%) in the control group. Of the 74 lesions with a depth of D1, the distribution by group was as follows: 25 (34%) in the infiltration group, 24 (32%) in the remineralization group, and 25 (34%) in the control group (Table 1).

### 3.4. Distribution by Lesion Activity

Activity could not be assessed in 62 (34.4%) lesions. The remaining 118 lesions (65.6%) were categorized as active—112 (95%) or inactive—6 (5%). According to the treatment method, the distribution of active lesions was very similar: 38 in the infiltration group, 37 in the remineralization group, and 37 in the control group. The distribution of inactive lesions was as follows: two in the infiltration group, three in the remineralization group, and one in the control group.

### 3.5. Comparison of the Treatment Groups on Lesion Progression One Year After the Treatment

**Figure 3:**
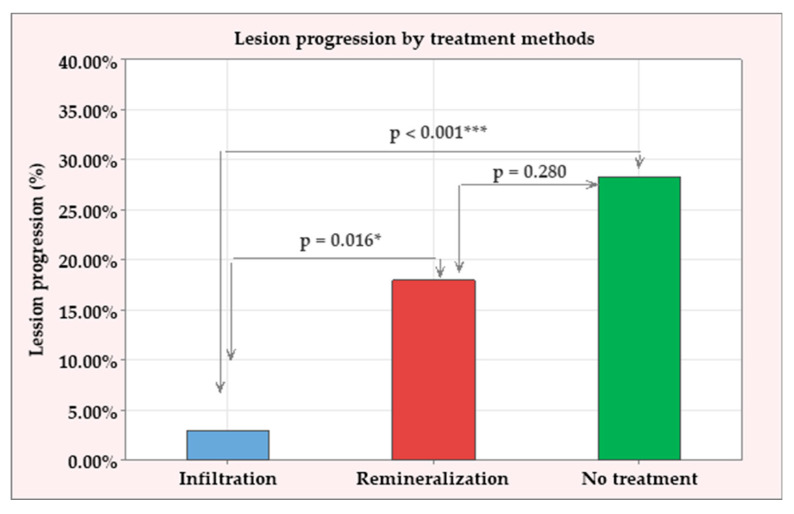
Progressive caries lesions one year after treatment by treatment method. *—Significant difference at level of significance of 0.05; ***—significant difference at level of significance of 0.001.

One year after treatment, the progression of caries lesions was assessed on bitewing radiography performed with the same X-ray holders, individual for each patient. Caries progression was observed in 30 out of all 180 lesions (16.6%), distributed across the three treatment groups: 2/60 (3%) in the infiltration group; 11/60 (18%) in the remineralization group; and 17/60 (28.30%) in the control (no treatment) group. The proportions of progressive caries lesions in each group were examined using a Chi-square test, which showed a significant difference between the treatments (*p* = 0.001). To specify these differences, post-hoc comparisons were conducted in pairs using Fisher’s exact test. The following results were identified (summarized in Figure 3):

1. The infiltration treatment showed a significantly lower percentage of progressive lesions than the other two groups (*p* = 0.016 compared to the remineralization group and *p* < 0.001 compared to the control group).
2. The remineralization group had a lower proportion of progressive lesions than the control group, but this difference did not reach statistical significance (*p* = 0.280).

**Figure 4:**
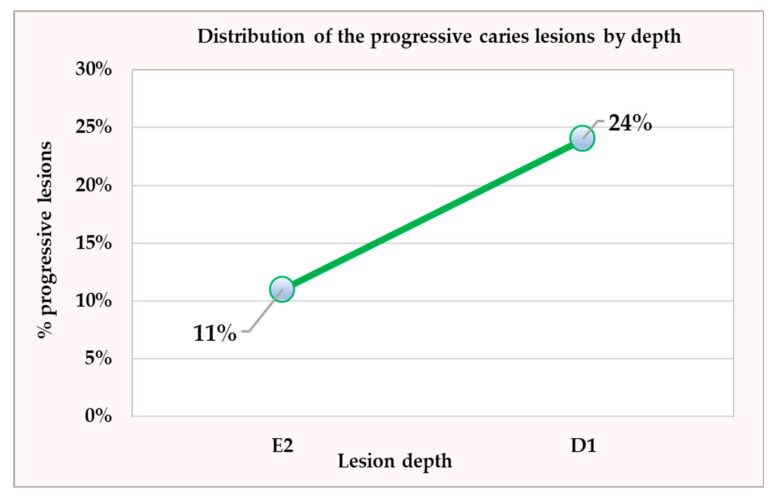
Distribution of progressive caries lesions related to lesion depth, where E2 is caries reaching the inner half of enamel, and D1 is caries reaching the outer third of dentin.

In terms of lesion depth, 12 progressive lesions (11%) out of a total of 106 were found at depth E2, and 18 progressive lesions (24%) out of a total of 74 were found at depth D1. There was a significant difference in the proportion of progressive lesions with depths E2 and D1, with the proportion of lesions with depth D1 being 13% higher than that of lesions with depth E2 (*p* = 0.037) (Figure 4).

**Figure 5:**
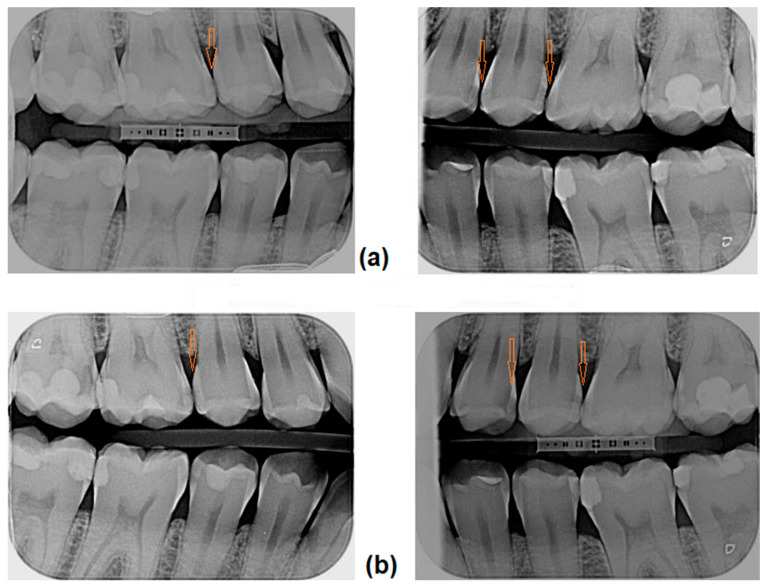
Bitewing radiographs before (**a**) and 1 year after treatment (**b**). Arrows indicate the initial proximal caries lesions and their condition at the 1-year follow-up. Tooth 16 mesially (caries D1)—infiltrated and arrested after 1 year. Tooth 25 distally (caries reaching DEJ)—remineralization, progressed after 1 year to caries D1. Tooth 24 distally (caries D1)—control (no treatment) progressed after 1 year to caries D2. E2 is caries reaching the inner half of enamel, D1 is caries reaching the outer third of dentin, and D2 is caries reaching the middle third of dentin.

Figure 5 demonstrates the bitewing radiographs of a patient before treatment and one year after treatment. Infiltrated lesions have been arrested, while remineralized and control lesions have progressed (Figure 5).

Bitewing radiographs were repeated 2 years after treatment, and the results were compared with those from the first year after treatment. Five new progressive lesions were identified, increasing the percentage of progressive lesions from 16.60% to 19.44% (an increase of 2.84%), though this change was not statistically significant (*p* = 0.584). According to the treatment applied, the distribution of the new progressive lesions was as follows: 0 in the infiltration group, 2 (13 in total) in the reminalization group, and 3 (20 in total) in the control group. Expressed in percentages, the dynamics of the lesions between the first and the second year after treatment was as follows: 0% in the infiltration group; 3.6% increase in patients treated with remineralization, from 18% to 21.6%, with no statistical significance of the change (*p* = 0.820); an increase by 5% in the control group, from 28,3% to 33.3%, without statistical significance (*p* = 0.693).

In terms of depth, the five new lesions included one with depth E2 and four with depth D1. There was a 1% increase in lesions with depth E2, rising from 11% to 12% (*p* = 1.00). The four new lesions with depth D1 represented a 5% increase, from 24% to 29%, but this was not statistically significant (*p* = 0.579). The results are summarized in Table 2.

In summary, there was a minimal increase in the number of lesions between the first and the second year, maintaining the trends reported in the first year after treatment. Significantly the lowest percentage of progressed lesions remained in the infiltration group (3%), followed by the remineralization group (21.6%) and the control group (33.3%), *p* < 0.001. The trend regarding the depth of lesions was also maintained, with a higher relative share of lesions with depth D1 (29%) compared to those with depth E2 (12%), *p* = 0.003.

## 4. Discussion

The present study indicates that resin infiltration of incipient proximal caries lesions is highly efficacious after one and two-year follow-up. The results confirmed the hypothesis of the clinical trial that lesion progression of infiltrated proximal caries was significantly reduced compared with non-infiltrated lesions receiving remineralization or no treatment. Lesion depth also demonstrated to be a significant factor for the risk of caries progression.

Radiographic progression of proximal caries in the enamel and outer third of dentin has been shown to be highest during adolescence and young adulthood [2]. The patients in the present study were predominantly in the latter group (aged 18–38 years). The positive effect of caries infiltration on arresting initial proximal lesions is consistent with other studies conducted on young patients [4] and children [29]. Follow-up with direct subtraction radiography after 18 months demonstrated the superior effectiveness of caries infiltration, with a success rate of 93% [10]. After 18 months, infiltrated lesions progressed in 7% of cases, compared to 37% of controls. After three years, progression was 4% for infiltrated lesions and 42% for controls [11].

In a study of young people at high risk of caries with a 12-month follow-up period, the effectiveness of caries infiltration was lower than in our study [29]. Apart from the reported higher caries risk, other factors may have contributed to this outcome [30]. Firstly, a greater proportion of D1 lesions were included in the study of children (60% compared to 41% in our study). Furthermore, the study was on deciduous teeth and did not use individual radiograph holders, increasing the risk of misreading caries progression [29]. The rate of caries progression in deciduous molars has been shown to be higher than in permanent premolars and molars, which may explain the lower infiltration efficiency observed in children. Other studies on deciduous teeth have also shown a higher rate of progression after infiltration than in our study: 40% for infiltrated lesions and 70% for control lesions [31], and 12% for infiltrated lesions and 33% for control lesions [4]. Lesion progression is quicker in primary teeth because the enamel structure is less mineralized, more porous, and thinner than in permanent teeth. Kabakchieva et al. compared the results of infiltrating proximal caries lesions in the permanent teeth of children with medium and high caries risk, reporting that the method was effective in arresting caries progression in patients with different levels of caries risk [32].

The results reported by Peters et al. on permanent teeth are very similar to ours: 3% of infiltrated lesions progressed, compared to 22% of controls [15]. Similarly, Arslan et al. found that 2.2% of infiltrated lesions progressed, compared to 20% of controls [14]. Studies on the long-term behavior of infiltrated lesions are limited. A 3-year follow-up trial reported 4% progression of infiltrated lesions and 48% progression of control lesions in patients with high caries risk [16]. Longer-term studies have also been reported: 18% progression of infiltrated lesions and 48% progression of control lesions after four years by Meyer-Lueckel et al. [33], and 9% progression of infiltrated lesions and 48% progression of control lesions after seven years of follow-up by Paris et al. [20].

The values cited in the literature vary considerably, but this is to be expected given the differences in the patient groups studied, their caries risk, the depth of the lesions (E2/D1), and the follow-up period. A common trend in all studies is that resin infiltration is a significantly more efficient treatment for initial proximal caries [34,35,36,37].

Infiltrating caries lesions is more effective for both primary and permanent teeth as compared to no treatment or other non-invasive preventive measures such as fluoride varnish, fissure sealant and oral hygiene instruction [37,38,39]. Remineralization was less effective than resin infiltration in the present study. Although remineralization produced better results than the placebo group, there was no statistically significant difference between them. This suggests that, while remineralizing agents can promote the arrest of initial caries, they cannot be recommended as a reliable sole treatment. These results are consistent with the literature data [5,39]. A positive, though insufficient, effect on inhibiting incipient caries lesions has been reported [5,6,40]. One possible reason is that remineralization of non-cavitated lesions is often limited to the superficial zone [8].

An individual Icon X-ray Holder (DMG, Hamburg, Germany) was used in this study to monitor infiltrated lesions and to ensure the same reproducible tooth and lesion position on each subsequent bitewing radiograph. This minimized the risk of misinterpreting the radiographic depth of the lesion due to proximal surface overlap and differences in lesion projection and geometry before treatment and during follow-up periods. Due to the lack of radiopaque material, infiltrated lesions cannot be distinguished from untreated lesions, either clinically or radiologically. Therefore, patients were given an identification card noting the treated and the control lesions. This allowed the examiner to rely blindly on control radiographs to determine the presence or absence of progression of various lesions, not knowing which lesion was involved.

The results of the study highlight the importance of lesion depth in determining caries progression risk and treatment selection. Lesions confined to the enamel (E2) demonstrated significantly lower progression rates compared to those extending into dentin (D1). This observation has its biological explanation: enamel lesions may be arrested or remineralized due to their high mineral content and lower organic component, whereas dentinal lesions are more porous and contain dentinal tubules that facilitate the inward movement of cariogenic acids and bacteria. Once dentin is involved, the lesion is not only structurally weaker but also more biologically active, creating an environment that favors progression despite preventive measures [19]. This underlines the necessity of early intervention before lesions reach the dentin–enamel junction and supports a stratified approach to treatment based on lesion depth [41]. Given its high efficacy, resin infiltration should be recommended as the first-line treatment for E2 lesions [10]. For D1 lesions, which involve dentinal structures and exhibited higher progression rates in this study, a more cautious or combined strategy may be proposed, especially for high-caries-risk patients. Infiltration alone may be insufficient due to the potential for micro-cavitation [34]. In such cases, clinicians could consider adjunctive measures, such as fluoride application, patient-specific risk management, shorter recall intervals, or even a minimally invasive restorative intervention if signs of lesion activity persist. Thus, at least postponing (if not avoiding) sacrifice of sound tooth structures could be achieved in case of more progressed treatment situations [3].

Patient compliance is also a crucial, though often underrecognized, factor influencing treatment outcomes in caries management [6,7]. All participants in this study received oral hygiene and dietary counseling. However, the fact that most of the progressed lesions were in the control and remineralization groups, where no mechanical seal was provided, highlights the importance of patient cooperation and the need for improved preventive strategies. In contrast, the effectiveness of resin infiltration may be less dependent on patient behavior, as it provides a physical barrier and mechanical stabilization of demineralized enamel to acid diffusion. The main results of the study support the philosophy of the early non-operative treatment of incipient caries lesions, which also emphasizes the need for proper case selection, regular follow-up periods, patient motivation, and re-motivation for oral hygiene and diet improvement.

Resin infiltration represents a micro-invasive treatment for initial caries lesions, filling the therapeutic gap between preventive approaches and operative caries management [3]. Its integration into routine clinical practice, however, requires the consideration of several practical factors. The procedure demands a precise technique which typically requires about 15 min per surface—more time-consuming than fluoride varnish application—and it remains significantly less invasive and less time-consuming than traditional restorative procedures. In terms of cost-effectiveness, resin infiltration is more expensive than varnishes, and insurance reimbursement may vary by region. Nevertheless, its potential to delay or eliminate the need for future restorative treatment could offer cost savings over the long term, particularly in moderate- to high-risk populations [10,18]. Considering patient experience, resin infiltration is highly acceptable. It is painless, non-invasive, and requires no local anesthesia or drilling, making it especially suitable for anxious patients and children [42]. Studies have demonstrated strong patient preference for infiltration over conventional fillings, citing reduced discomfort and greater aesthetic satisfaction [1].

### 4.1. Limitations

This study has several limitations that should be taken into consideration when interpreting the results. A 2-year follow-up period may not have been sufficient to assess the full extent of caries progression, particularly under preventive and non-invasive treatments that give their effects gradually. Another limitation is the use of bitewing radiographs alone to assess caries progression. While this imaging method is considered a gold standard for proximal caries detection in clinical settings, it is limited in its ability to detect subtle changes in lesion depth. Lesion progression assessed with digital subtraction radiography has been shown to be more accurate and to have better reproducibility compared with pair-wise comparison of radiograph, even though this technique has still not been widely adapted to the dental practice [10,43]. No adjunctive diagnostic tools such as optical devices (based on transillumination or fluorescence) were either used in the present clinical trial. In addition, the possibility of including lesions with micro-cavitations, particularly in lesions classified as D1, also represents a methodological concern. Despite careful clinical and radiographic examination, some lesions may have included undetected cavitations. Moreover, although all patients were given oral hygiene and dietary guidance, the variability in their compliance could have influenced lesion outcomes, especially in groups without a physical barrier such as the remineralization and control groups. Finally, the study involved a patient cohort of 47 individuals. While lesion-level analysis was adequately powered and included 180 sites, the findings may have limited generalizability to broader populations or different clinical environments.

### 4.2. Future Directions

Based on the findings of this study, several directions for future research could be outlined. Firstly, more long-term clinical trials are necessary, extending the follow-up period beyond two years to assess the long-term stability and durability of resin infiltration and remineralization treatments. Multicenter trials with more diverse patient populations (including children, adolescents, or patients with high caries and systemic risk) would improve validity. Future protocols could also incorporate non-ionizing technologies such as quantitative light-induced fluorescence, near-infrared transillumination, light and laser fluorescence, or optical coherence tomography to enhance lesion detection and monitoring. The use of harmless optical devices would allow more frequent and repeated control of lesion progression, as well as the inclusion of patients contraindicated for X-ray radiation like pregnant women and young children. Further comparisons between resin infiltration and new bioactive agents for enamel bioremineralization and regeneration (for example, self-assembling peptides) could identify more effective alternatives for non-invasive caries management. Last but not least, investigating the combined use of remineralization agents and resin infiltration may reveal synergistic effects, potentially expanding the indications for conservative management of initial caries.

## 5. Conclusions

1. Treatment of caries lesions by infiltration produced the best results after one and two years, with the lowest percentage of progressed lesions compared to remineralization treatment and the control group.
2. Remineralization was the second most effective treatment, showing a lower percentage of progressed lesions than the control group, though this difference was not statistically significant.
3. The control group with no treatment showed the highest percentage of progressed caries lesions.
4. The depth of the lesions significantly impacted the effectiveness of the treatment. A higher percentage of progressed caries was found in lesions with a D1 depth compared to those with an E2 depth.

In conclusion, resin infiltration can be considered an effective micro-invasive approach to arrest early proximal caries lesions.

## Figures and Tables

**Table 1:** Distribution of caries lesions by depth and treatment.

Treatment	Depth	
	Stage E2	Stage D1
Infiltration	35 (33%)	25 (34%)
Remineralization	36 (34%)	24 (32%)
No treatment	35 (35%)	25 (34%)
Total	106	74

**Table 2:** Change in the caries lesions between the first and second year after treatment by lesion progression, treatment, and depth.

Categories	1st Year	2nd Year	Change	*p*-Value
Total				
○ With progression	16.60%	19.44%	+2.84%	0.584
○ With no progression	83.40%	80.56%	−2.84%	0.716
By treatment				
○ Infiltration	3.0%	3.0%	0.0%	n.a
○ Remineralization	18.0%	21.6%	+3.6%	0.820
○ No treatment	28.3%	33.3%	+5.0%	0.693
By depth				
○ E2	11.0%	12.0%	+1.0%	1.000
○ D1	24.0%	29.0%	+5.0%	0.579

## Data Availability

The authors confirm that the data supporting the findings of this study are available within the article. Further inquiries can be directed to the corresponding author.

## Abbreviations

The following abbreviations are used in this manuscript:

E2	Caries reaching the inner half of enamel
D1	Caries reaching the outer third of dentin
DEJ	Dentin–enamel junction
TEGDMA	Triethylene glycol dimetacrylate
TCP	Tricalcium phosphate
